# Multivariate Analyses Applied to Healthy Neurodevelopment in Fetal, Neonatal, and Pediatric MRI

**DOI:** 10.3389/fnana.2015.00163

**Published:** 2016-01-21

**Authors:** Jacob Levman, Emi Takahashi

**Affiliations:** ^1^Division of Newborn Medicine, Department of Medicine, Boston Children's Hospital, Harvard Medical SchoolBoston, MA, USA; ^2^Athinoula A. Martinos Center for Biomedical Imaging, Massachusetts General HospitalCharlestown, MA, USA

**Keywords:** multivariate analysis, machine learning, fetal, neonatal, pediatric, brain MRI

## Abstract

Multivariate analysis (MVA) is a class of statistical and pattern recognition techniques that involve the processing of data that contains multiple measurements per sample. MVA can be used to address a wide variety of neurological medical imaging related challenges including the evaluation of healthy brain development, the automated analysis of brain tissues and structures through image segmentation, evaluating the effects of genetic and environmental factors on brain development, evaluating sensory stimulation's relationship with functional brain activity and much more. Compared to adult imaging, pediatric, neonatal and fetal imaging have attracted less attention from MVA researchers, however, recent years have seen remarkable MVA research growth in pre-adult populations. This paper presents the results of a systematic review of the literature focusing on MVA applied to healthy subjects in fetal, neonatal and pediatric magnetic resonance imaging (MRI) of the brain. While the results of this review demonstrate considerable interest from the scientific community in applications of MVA technologies in brain MRI, the field is still young and significant research growth will continue into the future.

## Introduction

The developing brain undergoes rapid structural and functional changes via a variety of processes including neuronal migration, axonal elongation, pruning, maturation of circuits and the emergence of convolution which support efficient signal processing regionally and among distant brain regions. Basic and higher-cognitive functions both require the coordination and cooperation of neurons located in multiple brain regions all of which are in a state of rapid development with a variety of different growth rates. Differences in brain activity between children and adults (Casey et al., 1997; Thomas et al., 2001; Bunge et al., 2002), the structural changes in many developing regions (Reiss et al., 1996; Gogtay et al., 2004; Fair et al., 2009; Supekar et al., 2009) which is linked to gradual changes in tissue contrast (Ketonen et al., 2004) and the recruitment of large cohorts of age matched subjects are major challenges facing researchers in fetal, neonatal, and pediatric imaging. Higher-order brain functions are supported by distributed patterns of brain activity and structure (Mesulam, 1981; Vaadia et al., 1995; McIntosh et al., 1996; Fox et al., 2005) and assessing and identifying these distributed patterns is particularly challenging in a pediatric/neonatal/fetal population due to small brain sizes, a rapidly changing physiology, a high degree of brain plasticity, patient motion, increased metabolism and an incomplete understanding of brain development.

Multivariate analysis (MVA) techniques (i.e., multivariate regression, multivariate analysis of variance, machine learning etc.) are advanced statistical, computational and pattern recognition technologies that evaluate multiple variables/measurements simultaneously. MVA technologies provide a theoretical improvement over univariate techniques which examine each acquired measurement individually. MVA has particularly large potential in studies of magnetic resonance imaging (MRI)-based brain development as many physiological and structural parameters can be measured, new measurements are constantly under development and distributed measurements across the entire brain are acquired. The ideal way to combine a distributed set of a variety of different physiological measurements for any particular application/investigation is not known *a priori*, making MVA research applied to the developing brain a challenging field of ongoing investigation. MVA techniques can be employed to discover what brain regions and what physiological biomarkers are most correlated with a variety of subject measurements such as sleep cycles in newborns, cognitive/psychological measures including the intelligence quotient (IQ) and language functions and lifestyle habits in older children. It is also possible to retrospectively correlate cognitive abilities (e.g., academic activities), lifestyle choices (e.g., smoking habits), or even pathogenesis of brain diseases exhibited later in life to developmental imaging examinations acquired during early brain development.

MRI provides a wide variety of different physiological measurements distributed across the brain, thus providing a wealth of information that may assist in an array of research problems in both clinical applications and basic research. The most common MRI modality produces basic structural information related to the concentration of hydrogen protons. Water is the most abundant molecule in the human body with two hydrogen protons found in each molecule. Since the body regulates many tissues and organs by controlling the concentration of water molecules across membranes, structural MRI provides excellent tissue contrast. Perfusion MRI measures blood perfusion by tagging fast moving hydrogen protons in the blood stream and monitoring the tissues to which they travel. Functional MRI (fMRI) measures a blood oxygen level-dependent signal which is associated with brain activity, an important method for monitoring brain function during an assigned task. fMRI can also be used to monitor normal blood oxygen levels in the brain while the subject is at rest. Diffusion weighted imaging (DWI) is focused on acquiring measurements of water diffusion which can be a useful physiological measurement in many applications. Diffusion tensor imaging (DTI) is a directional extension of DWI, measuring water diffusion in six or more different spatial directions while assuming a principal direction of water diffusivity at each pixel/voxel location in the brain. DTI allows the tracking of coherent tissue structures that are often associated with axonal and even glial fiber pathways (Takahashi et al., 2012; Xu et al., 2014) which has enormous potential for monitoring brain maturation in early developmental stages. MRI can acquire considerably more types of images as well that have not had a major impact in studies focused on imaging-based MVA of pediatric, neonatal, and fetal populations such as chemical exchange saturation transfer imaging (which includes pH sensitive amide proton transfer imaging) and MR spectroscopy (which is often not spatially resolved but a single measurement that is acquired across the entire brain or at a localized region-of-interest—ROI).

Recent years have exhibited remarkable growth in the use of MVA techniques in pediatric, neonatal and fetal imaging. An excellent review article on the use of MVA classification technologies in developmental brain imaging was previously published in 2009 (Bray et al., 2009), however, at the time of publication the number of research studies using MVA in a pediatric, neonatal and fetal population was limited. In the years since 2009, pre-adult brain MRI studies employing MVA technologies have exhibited remarkable growth, warranting a thorough systematic review. This article reviews MVA techniques applied to brain MRI of pediatric, neonatal and fetal populations and focuses on the imaging of healthy subjects.

## Materials and methods

### Multivariate analysis techniques

MVA techniques can be divided into several classes. Multivariate statistical techniques are quite varied in their potential applications, with a prominent example being techniques focused on the identification of measurements correlated with an important patient characteristic. With a large set of measurements available, MVA techniques such as multivariable linear regression (Rencher and Christensen, 2012) can be used to identify a subset of variables associated with a patient characteristic of interest. MVA techniques such as multivariate analysis of variance (MANOVA) (Warne, 2014) can help assess the effect of changes in one variable on dependent variables and can generally help elucidate the existing relationships between dependent and independent variables. Multivariate analysis of covariance (MANCOVA) (Smith, 1958) is a technique similar to MANOVA but can factor out noise or error introduced by a covariant variable. This review will discuss many applications of multivariate statistics in pediatric, neonatal and fetal populations without neurological/psychiatric disorders which will help illustrate the wide variety of potential uses of these techniques in a medical research context. Multivariate regression based techniques can also create new measurements that are a combination of existing measures creating customized factors/components associated with underlying physiological conditions. Principal Components Analysis (PCA) (Dunteman, 1989) is a representative example that computes orthogonal components that maximize the variance captured from the underlying measurements provided. Manifold learning performs dimensionality reduction non-linearly (Goldberg et al., 2008). Independent Components Analysis (ICA) (Hyvarinen et al., 2004) is a powerful technique based on discovering non-Gaussian distributions in datasets exhibiting mixed signals. These data reduction methods bridge the gap between statistical analysis techniques and related computational technologies that are used automatically and semi-automatically.

Machine learning (Carbonell et al., 1983) is a related class of analysis technique that exhibits considerable overlap with multivariate statistical approaches in that many also involve the selection of measurements and employ data reduction. However, machine learning is often considered a technology rather than a statistical analysis technique. Machine learning is divided into two main approaches: supervised and unsupervised learning. Supervised learning is a class of technologies that use training data which is a collection of measurements associated with multiple groups such as two different types of tissues of interest. The training data is used to inform future predictions, allowing the computer algorithm to assign new unknown samples to one of the groups for which it was provided example measurements. Some supervised learning algorithms include feature selection as part of the overall technology (the process of selecting measurements to rely upon for prediction), however, many do not and feature selection is often addressed as a separate topic in the scientific literature. Bayesian classification bases prediction on posterior probabilities computed from the distribution of training data provided (Devroye et al., 1996). The support vector machine (Vapnik, 1995) is a popular and high performing statistical machine learning technique that attempts to minimize the error on unseen samples and maximizes the margin that separates a decision function from the neighboring training samples provided. The relevance vector machine (Tipping, 2001) is an adaptation of the support vector machine that incorporates probabilistic Bayesian learning. The artificial neural network (Yegnanarayana, 2009) models the behavior of many neurons connected together in a wide variety of topologies to simulate the natural learning process exhibited in the brain. Linear discriminant analysis (McLachlan, 2004) computes a linear combination of measurements in order to characterize or separate two or more groups of samples. The decision tree is a simple methodology for embedding a series of decisions in a hierarchical structure and boosting trees (Friedman et al., 1998) is an adaptation that involves generating weights for imbalanced prediction or voting. The random forest (Breiman, 2001) is an additional extension of the decision tree in which a large collection of decision trees (dubbed a forest) are created with different predictive behavior allowing the algorithm classify a sample based on the most common predictions among those decision trees in the forest. The k nearest neighbor algorithm classifies a new sample based on the local data density of the provided training samples (Altman, 1992). Finally, the generalized linear model (Nelder and Wedderburn, 1972) is a flexible generalization of linear regression that allows for response variables with error distribution models other than the standard normal distribution. The generalized linear model is sometimes referred to as a multivariate statistical analysis technique, highlighting the considerable overlap between machine learning technologies and traditional statistical MVA techniques.

Unsupervised learning differs from supervised learning in that these MVA technologies are not provided with a set of example training data on which to base predictions. Instead, unsupervised learning technologies are tasked with performing a basic level of pattern recognition on a medical imaging examination based on the data in the examination itself. This typically involves dividing a medical examination into multiple regions-of-interest which can facilitate a variety of in depth analyses (this is also known as image segmentation). These technologies can be applied to isolating a particular tissue or structure in the brain and can be used to monitor changes due to healthy brain development. Unsupervised learning technology can support the extraction of regional physiological statistics and in turn can play a critical role in computer-aided diagnosis systems, supporting high-level patient-wide diagnoses. Example unsupervised learning technologies include the ISODATA algorithm (Ball and Hall, 1965) and cluster analysis (Manton et al., 2014). Cluster analysis is a family of techniques that includes hierarchical clustering in which data is structured across a hierarchical tree and also includes the k-means algorithm which finds k groups in a high dimensional dataset after random initialization. Graph cuts are formalized as an energy minimization problem (Greig et al., 1989). The Watershed method is inspired from geography and the image is modeled as a topographic map. The image is segmented based on ridge lines separating watershed catchment basins which represent regions-of-interest on the image (Beucher and Meyer, 1993). Also of interest is fuzzy set theory, which is used in situations where available information is incomplete or imprecise and functions by modeling uncertainty (Zadeh, 1965). Finally, the expectation maximization algorithm is a parametric approach that involves iterative refinement of parameter estimates (Dempster et al., 1977).

Several of the research papers included in this review made use of unsupervised learning algorithms and reported evaluative metrics for assessing the quality of the regions-of-interest produced by the learning technique. These metrics are reported in the results section of this paper and each of the relied upon evaluative metrics are introduced here. The Dice coefficient measures the amount of overlap between the computed region-of-interest (ROI) and ground truth data, defining that overlap as 2 times the magnitude of the intersect of the two ROIs divided by the sum of the magnitude of each individual ROI (Dice, 1945). The Jaccard index is closely related to the Dice coefficient though it assesses overlap as the magnitude of the intersection of the two ROIs divided by the magnitude of the union of the two ROIs (Jaccard, 1901). The Kappa statistic measures the agreement between raters by adjusting the relative observed agreement with the hypothetical probability of chance agreement (Cohen, 1960). For all three parameters, higher values indicate greater agreement between the gold standard ROI and the ROI computed by the learning procedure. Finally, Pearson's correlation coefficient (PCC) assesses the linear correlation between two variables. Highly correlated variables approach a PCC of 1, uncorrelated variables yield a PCC of 0 and highly negatively correlated variables approach a PCC of -1.

### Review parameters

The search engine MEDLINE/PubMed was used for this review on May 4th, 2015. The search terms employed were <Multivariate pediatric brain MRI> or <machine learning pediatric brain MRI> or <Multivariate neonatal brain MRI> or <machine learning neonatal brain MRI> or <Multivariate fetal brain MRI> or <machine learning fetal brain MRI>. This yielded 166 articles whose titles and abstracts were reviewed for their appropriateness for inclusion in this paper. Articles were excluded if brain MRI was not performed in a fetal, neonatal or pediatric population. Articles were excluded if they did not involve an MVA that included brain MRI data acquired as an important component of the analysis. Articles were excluded if not authored in English. Articles were excluded if they were not focused on healthy brains or normal neurological development. Articles from this search process that were not excluded were analyzed for this systematic review and noteworthy citations within these articles were considered for inclusion (subject to the same exclusion criteria).

## Results

**Figure 1:**
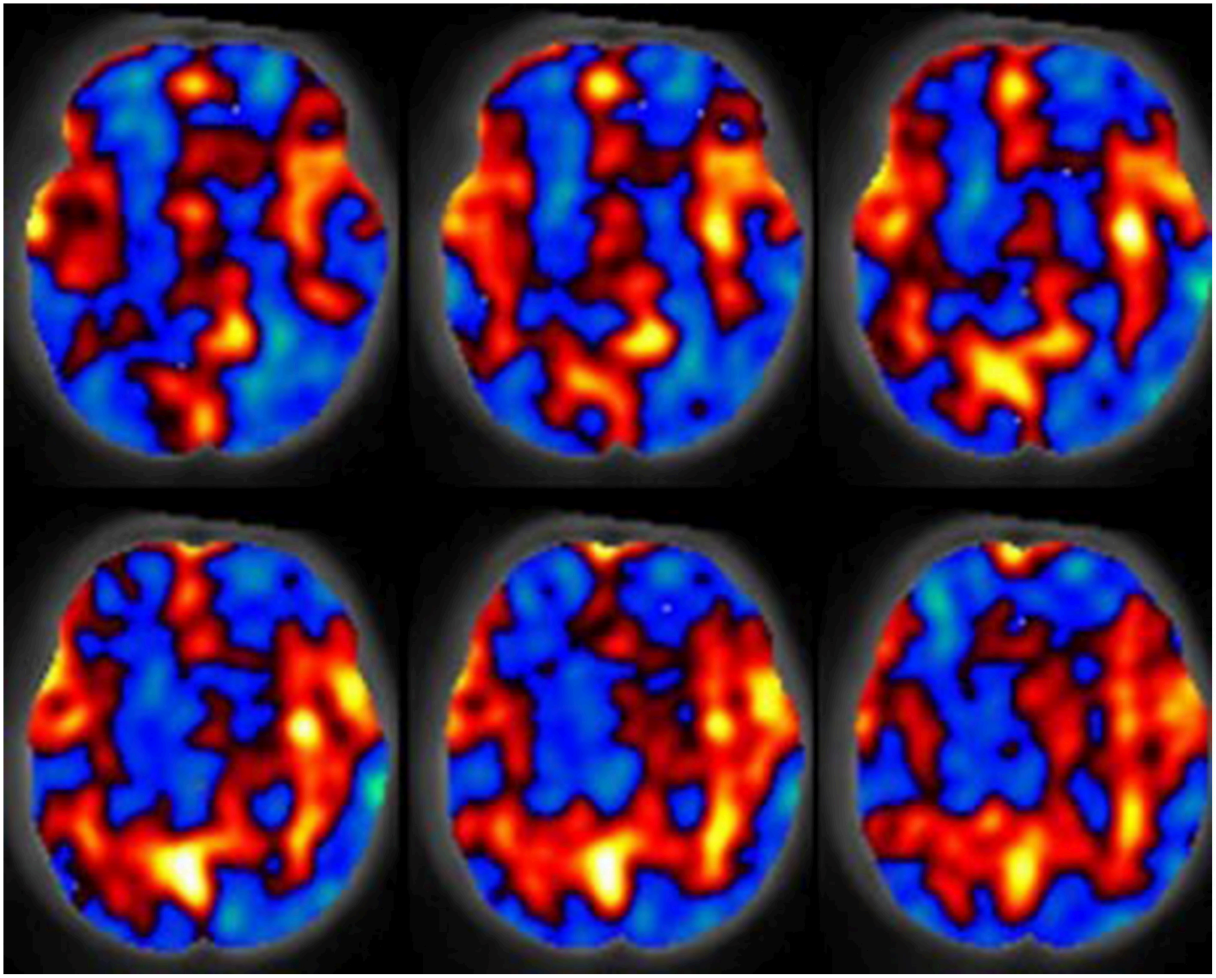
**The spatial relative cerebral blood flow discrepancy map comparing groups of subjects 7 and 13 months old**. Red values indicate greater blood flow in the 13 month group, blue values indicate greater blood flow in the 7 month group. Results were computed with a support vector machine. Figure is reproduced with permission (Wang et al., 2008).

**Figure 2:**
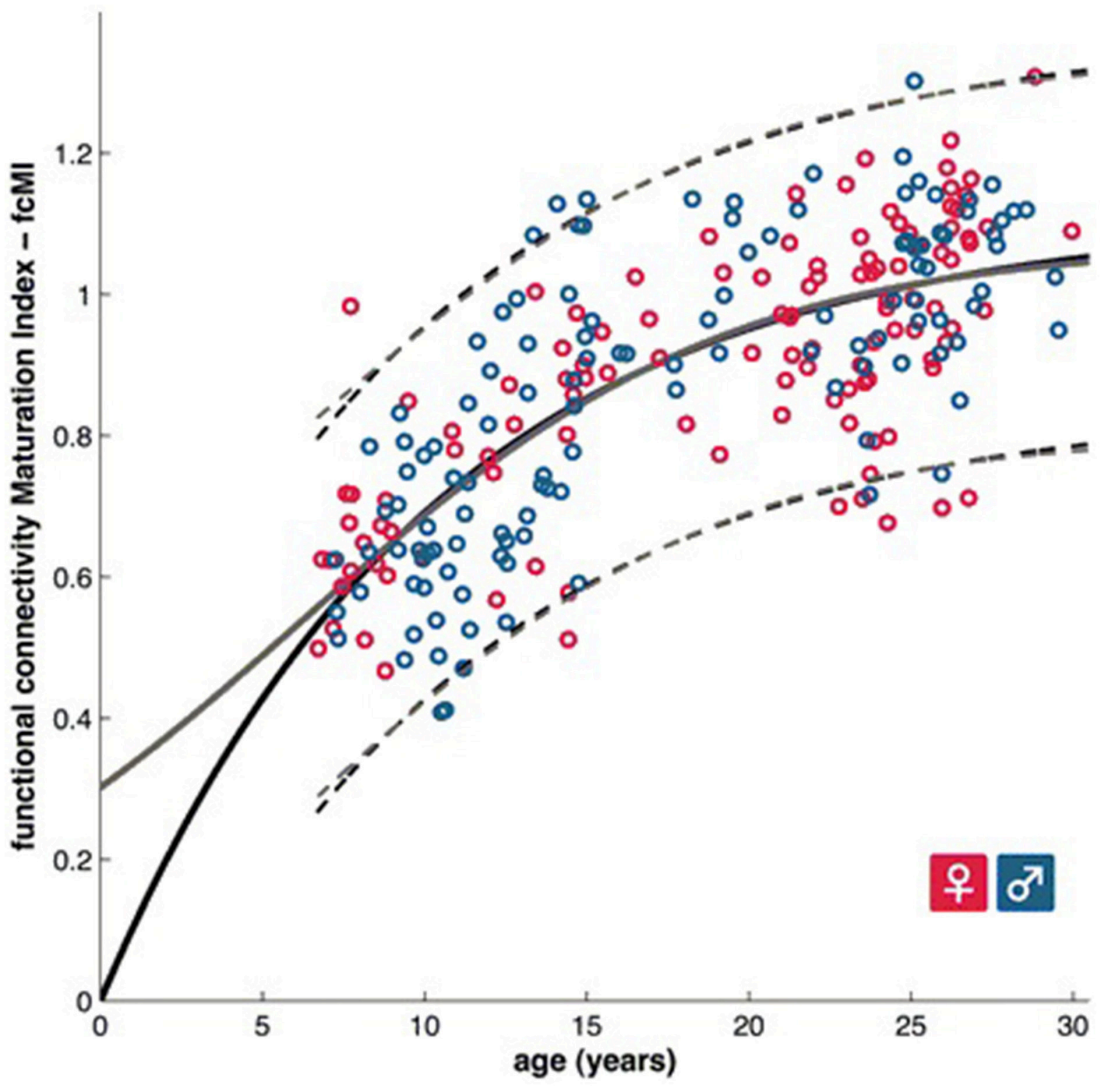
**A functional brain maturation curve comparing the functional connectivity maturation index as computed with multivariate techniques with subject age (Dosenbach et al., 2010)**. Figure is reproduced with permission.

**Figure 3:**
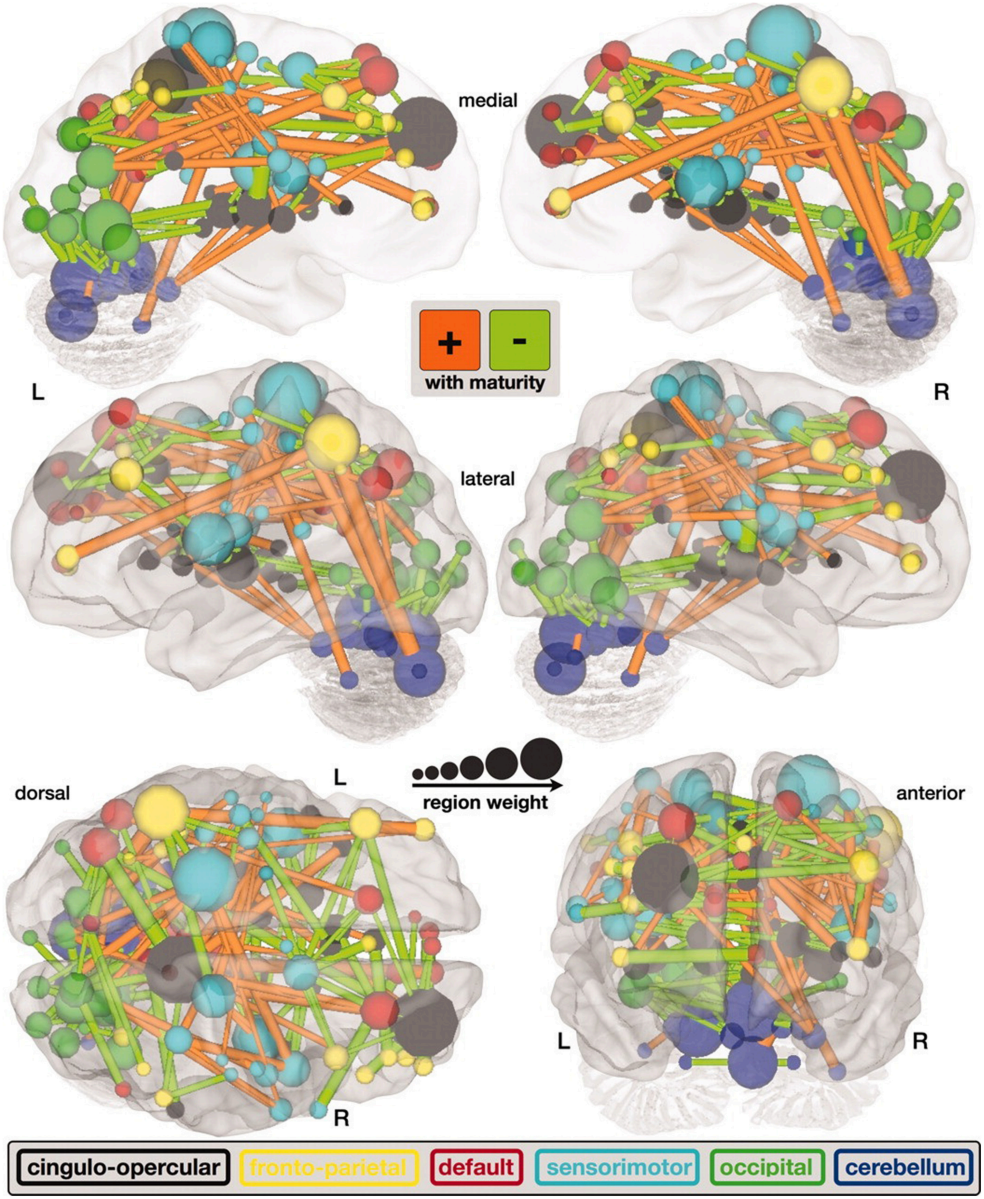
**A map of functional connectivity in the brain computed with the support vector machine**. Connections positively correlated with age are shown in orange. Negative correlations with age are shown in light green (Dosenbach et al., 2010). Figure is reproduced with permission.

**Figure 4:**
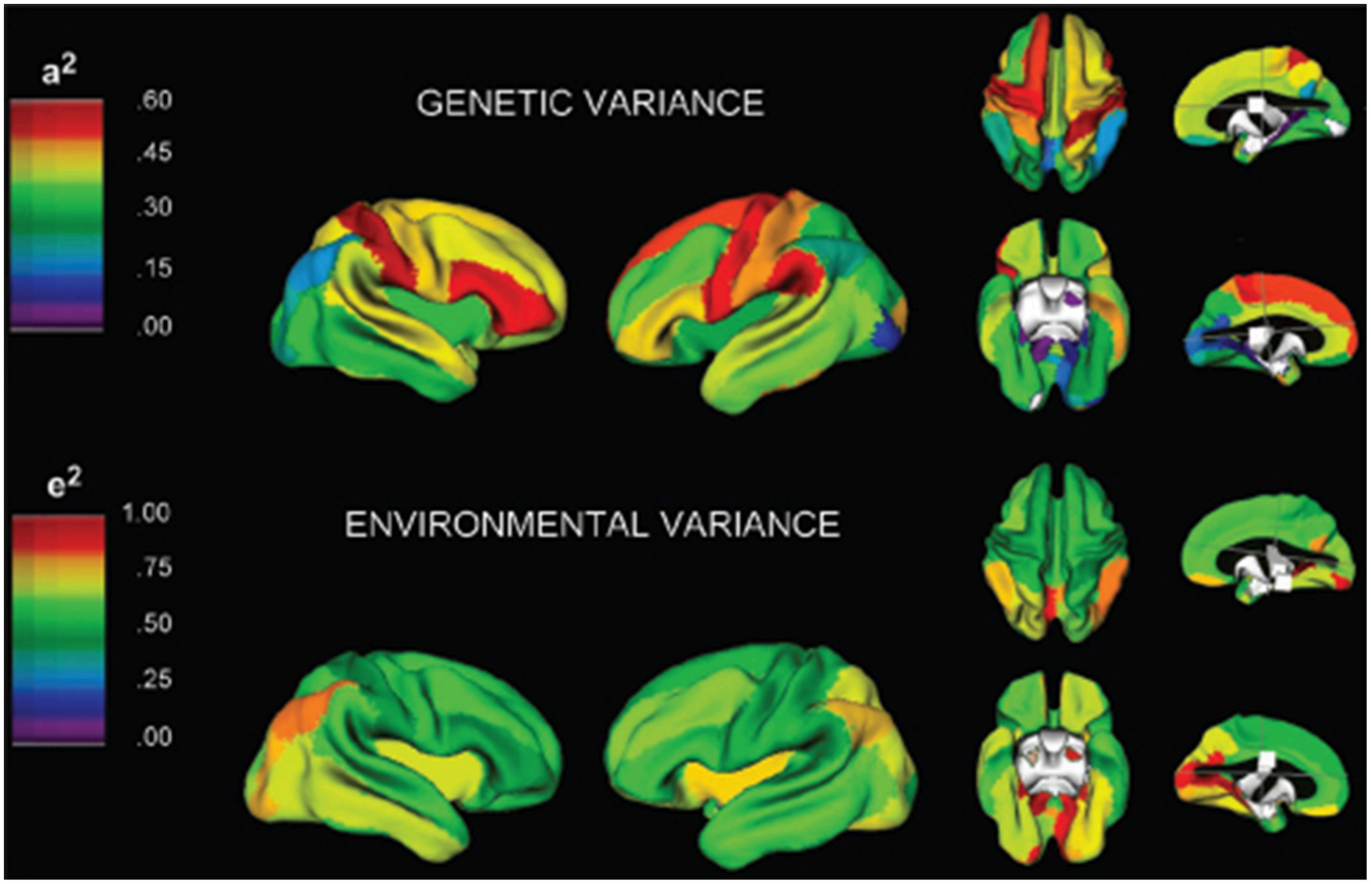
**A three-dimensional rendering of the brain with overlaid color maps illustrating the relative contribution to variability of different neurological locations based on genetic and environmental factors (Schmitt et al., 2007)**. Figure is reproduced with permission.

At present we have an incomplete understanding of healthy human brain development and the results of this review demonstrate that MVA techniques combined with MR imaging can play a substantive role in helping elucidate our knowledge pertaining to developmental brain imaging. In addition to enhancing our fundamental knowledge, theoretically, a more complete understanding of healthy human brain development may lead to the accurate identification of deviations from expected neurodevelopment. This in turn may assist in the detection, characterization, diagnosis and progression monitoring of a wide variety of medical disorders as aberrations from healthy brain development. MVA technologies can be used to assist in understanding, characterizing, and monitoring healthy brain development. MVA technologies based on MRI can be used to create tools to assist in the analysis of the images we acquire such as assessing gray and white matter volumes, detecting structural changes during development, identifying anatomical substructures associated with a phase of brain development and predicting a subject's age or gender. MVA provides considerable potential over traditional univariate analyses with a wide variety of flexible applications of MVA available. MVA can be used to generate a custom index that combines multiple measurements (as in Figures 1, 2). Additionally, MVA can be used to visualize the extent of tissue connectivity (as in Figure 3). MVA can also be used to create images that allow for visual comparison of regional differences in group-wise analyses (see Figures 1, 4).

### Patterns of brain development

MVA technologies can be applied to brain MR imaging data to help identify patterns associated with a wide variety of aspects of healthy brain development. Studying human brain development is first possible with fetal imaging, which allows the assessment of a subject's neurodevelopment *in utero*. Schopf et al. (2012) investigated a fetal population with resting state fMRI of the brain using ICA and demonstrated that resting state functional networks in the fetal brain are detectable *in utero*. Their study looked at a population observed across gestational weeks (GW) 20–36 and found three important network components associated with this developmental stage: a bilateral frontal, a bilateral occipital and a unilateral temporal component located in the left hemisphere. Thomason et al. (2013) studied a healthy fetal population with resting state fMRI also making use of ICA for analysing functional networks of activation in the brain. ICA identified eight bilateral networks. ICA was also able to isolate five components associated with noise, emphasizing the technique's ability to not only identify regions of the brain with similar activation patterns, but also to separate noise from imaging datasets which can assist in improving the signal-to-noise ratio (SNR) of the MR examinations. Jakab et al. (2014) investigated the use of fMRI for *in utero* imaging of the fetal brain. Their work used PCA-based feature measurement reduction as a means of removing noise from the imaging data to support more reliable analyses. Results demonstrated that the overall connectivity network in the brain as well as short range and interhemispheric connections exhibited a sigmoid expansion curve which peaked at 26–29 GW. By contrast, long range connections exhibited a linear increase with no periods of peaking development. Their results demonstrate heterogeneous development of functional networks in the fetal brain. Ferrazzi et al. (2014) presented a framework for improving fetal MRI studies that make use of ICA by correcting for imaging artifacts induced by motion, bias field and spin history. Their results were found to be consistent with identified resting state functional networks reported in previous fetal imaging studies.

Children born healthy but prematurely or with very low birth weight represent an interesting research group which does not necessarily have neurodevelopmental disorders. This group provides unique opportunities for study as they represent an intermediary between fetal and standard neonatal imaging. Tich et al. (2011) applied multivariable regression to data from very preterm infants and demonstrated that larger birth weight, shorter duration of assisted ventilation and older postmenstrual age (gestational age at birth + postnatal period) at MRI were predictive of larger brain metrics. Furthermore, biparietal diameter was found to be the variable most associated with the Mental Development Index and the Psychomotor Development Index. Nosarti et al. (2004) investigated the size of the corpus callosum among very preterm birth children and its relationship to neuropsychological outcome. Their analysis included MRI and MANCOVA. Their results indicated that in preterm boys only, verbal IQ and verbal fluency scores were positively associated with total mid-sagittal corpus callosum size and mid-posterior surface area. Adams et al. (2010) investigated the use of diffusion tractography in premature newborns and applied multivariate regression which indicated that gestational age at birth was not significantly associated with DTI measures of corticospinal tract development. Fearon et al. (2004) investigated the long-term effect on the brains of very low birth weight newborns by imaging them with MRI during adulthood and analysing the results with the general linear model (GLM). The GLM demonstrated that ventricular volume was larger and posterior corpus callosum volume was smaller in very preterm individuals compared with controls. Delpolyi et al. (2005) investigated the microstructural and macrostructural development of the cerebral cortex in premature newborns using DTI and traditional statistical multivariate techniques. Cortical gyration was measured as the ratio of gyral height to width on volumetric MRI bilaterally in the superior frontal, superior occipital, precentral, and postcentral gyri. Although, cortical gyration scores, fractional anisotropy and radial diffusivity were all significantly correlated with the estimated gestational age, MVA found no statistically significant relationship between DTI parameters and cortical gyration beyond their common association with estimated gestational age.

Imaging of neonatal brain development is a challenging task as the brain is relatively immature and exhibits a reduction in organized neuronal activity as compared to adults. The newborn brain is also maturing rapidly both structurally and metabolically. Aljabar et al. (2010, 2011) demonstrated that MVA based manifold learning technology can be applied to the developing neonatal brain and has potential toward identifying patterns in the trajectories of brain development. Their results demonstrated a strong correlation between clinical data such as gestational age, weight and head circumference with multivariate MRI-based measurements. Furthermore, their approach was shown to produce improved correlations with subject age over measurements extracted from MR examinations. Wang et al. (2008) demonstrated that blood perfusion MRI can be used in combination with the multivariate technique known as the support vector machine to analyse cerebral blood flow increases in the hippocampi, anterior cingulate, amygdalae, occipital lobes, and auditory cortex demonstrating increased cerebral blood flow in 13 month old infants relative to a 7 month old group. Their results also demonstrated decreased blood perfusion in the right temporal lobe, right prefrontal region and the left putamen. Figure 1 is provided to demonstrate relative cerebral blood flow discrepencies between the 13 and 7 month old groups as computed by a support vector machine. Matsuzawa et al. (2001) studied age-related volumetric changes in both gray and white matter in healthy infants and children. Their analysis included the use of a Bayesian algorithm to assist in characterizing gray matter, white matter and cerebrospinal fluid from MRI examinations. Their study also included a MANOVA statistical analysis. Their results helped quantify natural growth spurts occurring during the first 2 years after birth, a period during which the frontal lobes grew more rapidly than the temporal lobes. Right-left hemispheric asymmetry was more noticeable in the temporal lobes than in the frontal lobes. White matter volume was shown to increase at a higher rate than gray matter volume throughout childhood. Koshiba et al. used principal component analysis to examine factors associated with the neurological and behavioral development of “Head Control” and “Roll Over” in a neonatal population (Koshiba et al., 2015). They determined that hematological and brain anatomical factors were correlated with these basic neonatal movement patterns.

Imaging of children that have grown beyond the neonatal phase is another important aspect of neurodevelopmental brain imaging. These pediatric populations are less challenging to image than fetal and neonatal populations due to a variety of factors including a larger nervous system and some degree of cooperation from the subjects regarding remaining still during imaging (movement can corrupt the examination with motion artifacts). Giedd et al. (1999) presented a landmark longitudinal study which helps to introduce this topic. Their analysis included 145 healthy subjects between 4 and 20 years old. Their study incorporated an artificial neural network for tissue classification. Their results demonstrated linear increases in white matter and nonlinear changes in cortical gray matter that varied by brain region. Shaw et al. (2008) presented a large-scale cortical thickness study investigating 764 MRI examinations acquired longitudinally from 375 typically developing children and young adults. They used regression analysis to determine if each cortical measurement was best modeled by a cubic, quadratic, or linear function as they vary with age. They determined that most of the lateral frontal, lateral temporal, parietal, and occipital isocortex developed with a cubic trajectory exhibiting a period of initial childhood increase followed by adolescent decline and then stabilization. The quadratic model exhibiting a period of initial childhood increase followed by a decrease of cortical thickness without a period of stabilization was identified in the insula and the anterior cingulate cortex. A linear growth trajectory was observed in the posterior orbitofrontal and frontal operculum, portions of the piriform cortex, the medial temporal cortex, subgenual cingulate areas, and medial occipitotemporal cortex. Chen et al. (2014) investigated a combination of neonates and children up to 4 years old helping to bridge the gap between neonatal and pediatric imaging analysis of brain development. Their study used multivariate adaptive regression splines to derive data-driven growth trajectories for the three eigenvalues (measures along three principal directions of water diffusivity) associated with DTI and demonstrated that insights into brain maturation can be gained through analysing eigenvalues. Specifically, their work revealed limitations in relying upon the average of the secondary and tertiary eigenvalues for radial diffusivity because they exhibited significantly different growth velocities compared to that of the first eigenvalue. Based on the three primary eigenvalues, their results also demonstrate growth trajectory differences between the central and peripheral white matter, between the anterior and posterior limbs of the internal capsule and between the inferior and superior longitudinal fasciculus. Schmithorst et al. (2007) investigated the development of effective connectivity pertaining to narrative comprehension in children aged 5–18 using fMRI, independent components analysis and the general linear model. Feedback networks were identified during a narrative processing task involving effective connectivity from Broca's area and the medial aspect of the superior frontal gyrus to the posterior aspects of the superior temporal gyrus bilaterally. They also demonstrated that the effective connectivity from Broca's area to the superior temporal gyrus in the left hemisphere increases with age. The results demonstrate that it is feasible to investigate effective connectivity using MVA applied to multiple subjects in the absence of an *a priori* model. In their analysis functional activation maps in the brain were computed with the general linear model.

### Predicting brain age/maturity

Predicting brain age/maturity from MRI examinations has the potential to play an important role in both improving our understanding of healthy brain development and studying the nature by which developmental disorders deviate from expectation. Brown et al. (2012) studied healthy brain development with structural MRI and multivariate regression and demonstrated that their model of human brain maturation accounted for over 92% of the individual variability in brain development as defined by subject age. Mwangi et al. (2013) used the relevance vector machine combined with diffusion tensor imaging demonstrating that they could produce an index closely related to subject age (with Pearson correlation coefficients ranging from 0.870 to 0.899 depending on the DTI measurement relied upon). Brain maturity assessment was demonstrated to be feasible based on combining resting state fMRI with support vector machine technology in subjects aged 7–30 years old (Dosenbach et al., 2010). The greatest contribution to predicting brain maturity was based on the weakening of short-range functional connectivity between the brain's major functional networks, consistent with the work by Jakab et al. (2014). However, it should be noted that the work by Power et al. (2012) calls into question fMRI findings of a shift from short to long range connectivity in the presence of patient motion artifacts which can bias fMRI based short and long range connectivity analyses. Figure 2 provides a functional maturation index computed with the aid of multivariate techniques relative to age demonstrating how MVA can be used to help better understand and characterize brain development (Dosenbach et al., 2010). The resultant functional maturation curve accounted for 55% of the sample variance following a nonlinear asymptotic growth curve shape. Figure 3 provides a connectivity map demonstrating functional connections as computed with the support vector machine (Dosenbach et al., 2010). Greene et al. (2014) utilized the support vector machine to reliably classify individuals as children or adults based on basal ganglia cortical system functional connectivity yielding an accuracy of 83.3%. Smyser and Neil (2015) demonstrated that they could use the MVA technique known as the support vector machine to demonstrate differences between term and very preterm infants based on resting state functional MRI examinations. Franke et al. (2012) demonstrated that the relevance vector machine combined with MRI could accurately predict the age of the brain being analyzed. Furthermore, they demonstrated that preterm-born adolescents exhibited a significantly lower estimated brain age than their chronological age. Correlations between subject age and that estimated by their approach ranged from 0.9 to 0.95 and represents a statistically significant finding. Serag et al. (2012) employed unsupervised learning technologies and reported that their approach can produce a good biomarker of brain development. Toews et al. (2012) presented a model that can be used to identify age-related anatomical structure using Bayesian techniques to predict the age of subjects with an average error of only 72 days. Khundrakpam et al. (2015) presented an approach to assessing brain maturity based on cortical gray matter thickness and a linear regression model and found that the leading predictors were highly localized sensorimotor and association areas. An approach to estimating brain age based on DTI was presented by Han et al. (2014) at conference. Dittrich et al. (2014) used a random forest classifier to construct an atlas of fetal brain development which was used to estimate a brain structure's age morphologically. Erus et al. (2015) demonstrated that multivariate regression can be used to model healthy structural brain maturation and showed that deviations from expectation are correlated with cognitive performance for both developmentally delayed individuals as well as those with cognitive precocity. Results demonstrated that the brain development index that they present is correlated with subject age with a correlation coefficient of 0.89.

### Identifying brain and tissue structures

MVA technologies can be used to perform pattern recognition on a pediatric patient's brain MRI examination in order to divide the image into regions-of-interest (ROIs) separating tissues and structures for further analysis, a process referred to in the literature as image segmentation. Such techniques establish a set of ROIs in the examination which can facilitate studies investigating changes in white and gray matter volumes, structural changes, and tissue outcome prediction studies. These techniques can be used as an important component in a computer-aided detection system which can assist in the characterization and identification of a variety of complex regional variations of brain development. This subsection of the results first presents MVA studies applied to a fetal population followed by neonatal and then pediatric populations.

The germinal matrix is a transient deep brain region of developing cells adjacent to ventricles that is present in the fetal brain between 8 and 28 weeks gestational age. Habas et al. (2008) presented an approach to the segmentation of the germinal matrix from *in utero* clinical MRI examinations of the fetal brain at conference. Their approach was based on a constructed tissue atlas formed by a combination of subject MR examinations which yielded average shape and intensity images. Keraudren et al. (2013) developed a technique using MRI to detect the location of the fetal brain from the mother's abdomen and applied it to a database of 59 fetal examinations and also applied their segmentation approach to motion correction (Keraudren et al., 2014). Their work was based on fetal T2 weighted structural MRI.

Altaye et al. (2008) developed infant brain probability templates for MRI segmentation and normalization. Their approach was based on creating tissue probability maps from 76 infants ranging in age from 9 to 15 months. They demonstrate the utility of their approach by segmenting imaging examinations into gray matter, white matter and cerebrospinal fluid. Commowick and Warfield (2010) used an expectation maximization approach to support the segmentation of neonatal brain MRI examinations using T1 and T2 structural imaging. Shi et al. (2010) created an approach to neonatal brain image segmentation based on probabilistic atlases. The authors reported Dice coefficients (range 0.85–0.87) that demonstrated that their approach has similar agreement to the results of two experts. He and Parikh (2013) developed a brain segmentation technique based on T2 relaxometry and demonstrated their work on very preterm infants yielding a Dice coefficient of 0.95.

Weisenfeld and Warfield (2009) presented an approach to the probabilistic segmentation of each pixel/voxel from spatially aligned T1 and T2-weighted neonatal MRI examinations of the brain into cortical and subcortical gray matter, unmyelinated white matter, myelin, cerebrospinal fluid, and background (non-brain). Their approach shares similarities with Bayesian techniques and was reported to have achieved an accuracy comparable to that obtained by semi-automatic methods that require manual interaction from the user (with average Dice coefficients ranging from 0.72 to 0.92 depending on tissue type).

Song et al. (2006) developed an approach to the segmentation of T2 MRI examinations of neonates based on graph cuts technology incorporating a new method for information integration. Tissue priors and local boundary information are integrated with standard image intensity values into the edge weight metrics used by graph cuts. Their approach also incorporated inhomogeneity correction. They demonstrated that their method outperformed a commonly used optimization method applied to segmentation.

Xue et al. (2007a) developed an automatic approach to cortical segmentation in the developing brain that uses *a priori* knowledge of the inverted contrast exhibited between gray and white matter when myelination is incomplete. Their approach was tested on T2 imaging examinations from 25 neonates and compared with the results of manual segmentations. In an additional study, Xue et al. (2007b) focused on the segmentation and reconstruction of the neonatal cortex from T2 MRI examinations using an expectation maximization Markov Random Field approach. Their results indicated that cortical surface area and curvature increase with age. They determined that whole brain surface area scales to cerebral volume according to a power law while cortical gray matter thickness is not related to age or brain growth. They report Dice coefficients of 0.76 for gray matter and 0.79 for white matter. Isgum et al. (2015) presented the results of the NeoBrainS12 challenge which involved eight participating research teams attempting to segment preterm neonatal T1 and T2 MRI brain examinations into cortical gray matter, non-myelinated white matter, brainstem, basal ganglia and thalami, cerebellum, and cerebrospinal fluid in the ventricles and in the extracerebral space. Teams involved in the competition took a variety of multivariate approaches including one based on watershed segmentation, three techniques based on the k nearest neighbor algorithm and four techniques based on expectation maximization. An implementation of k nearest neighbor yielded the highest Dice coefficients (0.56–0.95 depending on tissue type) on axial images. Analysis of the results of the competition indicated that the automatic segmentation of brain tissues from neonatal MRI examinations is feasible, however, the automatic segmentation of myelinated white matter in these images is not feasible (with a Dice coefficient of 0.56 corresponding to the segmentation of myelinated white matter).

Zhang et al. (2015) developed a neonatal brain segmentation system using artificial neural networks based on multiple MRI modalities achieving Dice coefficients of 0.83–0.86 depending on tissue type. Wang et al. (2015) presented an approach to neonatal brain segmentation based on the random forest supervised learning algorithm yielding Dice coefficients ranging from 0.83 to 0.92 depending on tissue type. Song et al. (2007) presented an approach to neonatal brain segmentation based on Bayesian analysis and the support vector machine and published their work at conference. A level-set based brain extraction technique was developed by Shi et al. (2012) and applied to both neonatal and pediatric examinations yielding Jaccard indices of 0.95–0.96 depending on subject age. Devi et al. (2014) presented an approach to automatic brain segmentation of neonates employing atlas based probabilities and Gousias et al. (2012) also employed probability based atlases but their approach was applied to a pediatric population yielding Dice coefficients ranging from 0.9 to 0.92 depending on the brain's substructure being evaluated.

The segmentation of the pediatric brain has been the subject of numerous studies. Glass et al. (2003) developed an approach to the prediction of total cerebral tissue volumes based on multimodal MRI examinations (T1, T2, proton density, FLAIR). Their approach employed a hybrid artificial neural network segmentation and classification algorithm used to identify normal parenchyma. They reported an average error of estimation of total cerebral tissue volumes of 6% in 27 min of computational processing time or alternatively an average error of less than 2% based on 2 h and 4 min of processing time. Shan et al. (2006) developed a brain atlas based on the structural T1 volumetric MRI examination of a 9-year-old girl, involving the construction of a three-dimensional triangular mesh model and indicated that their results could be used to plan treatment, conduct model-driven segmentation and to analyze the shapes of brain structures in pediatric patients. They reported kappa statistics of 0.97 for cortical regions and 0.91 for subcortical regions indicating substantial similarities between their mesh model and the original volumes.

The segmentation of the caudate nucleus from pediatric (aged 2–4 years) MRI examinations was the subject of a component of a grand challenge as part of a workshop associated with the *Medical Image Computing and Computer Assisted Intervention* Conference (van Ginneken et al., 2007). The competition entrants included Arzhaeva et al. (2007) who presented a method based on the k-nearest neighbor classifier. Wels et al. presented a method (Wels et al., 2007) based on probabilistic boosting trees. A multiple atlas based approach was presented by van Rikxoort et al. (2007) and a probabilistic atlas approach was presented by Gouttard et al. (2007). Levy et al. (2007) presented an approach based on Bayesian optimization. Babalola et al. (2007) presented an approach based on active appearance models. Tu et al. (2007) presented an approach based on hybrid generative/discriminative models. Schonmeyer and Schmidt (2007) presented an approach combining pixel-based and object-based measurements derived from cognition network technology. Finally, Liu et al. (2007) presented an approach based on active contour models. There were reporting differences between the studies that entered the competition, making direct comparisons of their performance on the pediatric population challenging. However, of those studies that reported a Pearson correlation coefficient specific to the pediatric population included in the competition, Babalola et al.'s results (Babalola et al., 2007) were the most accurate (Pearson coefficient: 0.8320).

### Shape analysis

Shape analysis typically involves a statistical approach to taking measurements from images indicative of a brain structure's morphological properties such as how spherical, irregular or elliptical a region presents on MRI. Shape measurements are typically extracted from ROIs established through image segmentation (see previous subsection). Shape information can be useful for assessing healthy growth patterns and theoretically can assist in creating measurements that may be able to identify aberrations from healthy growth trajectories. In terms of healthy brain development, Batchelor et al. (2002) proposed an MVA approach to studying the shape of the cerebral cortex using a set of measurements useful to assist in quantifying folding in the brain from fetal MRI examinations. Their study focused on the imaging of *ex vivo* brain specimens which included a wide variety of pathological findings. Their subjects included a sample that was the result of spontaneous miscarriage without pathological findings. Rodriguez-Carranza et al. (2006) developed a system for measuring regional surface folding in neonatal brain MRI examinations, allowing evaluation of surface curvature within subregions of the cortex. Their method was applied to seven premature infants born at 28–37 gestational weeks and gray matter and gray-white matter interface surfaces were extracted. Their research can theoretically support the study of structural development in the neonatal brain within specific subregions. Serag et al. (2012) created a system capable of performing unsupervised learning of shape complexity and reported that their approach can produce a good biomarker of brain development.

### Studying gender in the brain

Comparing gender differences based on MRI examinations is a common technique used to help elucidate developmental variability between the sexes. There have been a few studies focused on the analysis of gender differences in healthy subjects using MVA techniques. Casanova et al. (2012) used the random forest algorithm to investigate gender differences based on resting state functional imaging yielding a classification accuracy of 65%. They concluded that gender differences may be related to regional connectivity differences between critical nodes. Awate et al. (2010) developed a multivariate modeling approach to the analysis of cerebral cortical folding and demonstrated its utility in studying cerebral cortical folding differences between genders. Skiold et al. (2014) studied boys and girls born extremely preterm with MRI using the generalized linear model and determined that cognitive and language outcomes at 30 months were poorer in boys.

### Relating genetics to brain MRI

Genetics provides a wealth of information about subjects studied. Given that there is still limited knowledge on the functional role and organization of the human genome, it is expected that genetic analyses will continue to grow in importance. There have been several studies to date focused on combining genetic data with MR brain imaging and also making use of MVA techniques. Giedd et al. (2007) investigated the relative impact of genetic and environmental factors on human brain anatomy during childhood and adolescent development by applying multivariate analysis techniques used in genetic analyses to a large sample of monozygotic and dizygotic pediatric twin's MRI examinations. Their results indicated that cross twin correlations were substantially higher in the monozygotic group relative to the dizygotic group, indicative of a strong genetic impact on brain volume variations. Although, their results indicated that environmental factors provided a much smaller contribution to brain volume variations (both whole brain and in specific subregions), environmental factors did influence a substantial portion of the variance in the dataset.

Schmitt et al. (2010) demonstrated that in a large pediatric population most of the variance in the brain's substructures is associated with highly correlated lobar latent factors with differences in genetic covariance and heritability driven by a common genetic factor that influences white and gray matter differently. In another study Schmitt et al. (2009) developed a novel method that combines classical quantitative genetic methodologies for variance decomposition with semi-multivariate algorithms for high resolution measurement of phenotypic covariance assessed with structural T1 MRI examinations. Their results indicated that mean cortical gray matter thickness was most strongly correlated with genetic factors in association cortices. Their study suggests that genetics plays a large role in global brain patterning of cortical gray matter thickness. In an additional contribution, Schmitt et al. (2008) employed principal components analysis of the genetic correlation matrix and structural MRI examinations of pediatric twins and siblings. Their results identified genetically mediated fronto-parietal and occipital networks. Figure 4 provides a three-dimensional rendering of the brain with overlaid color maps which illustrate the relative contribution to the variability seen in different neurological locations based on genetic and environmental factors as computed with the aid of multivariate techniques. In yet another contribution, Schmitt et al. (2007) analyzed MRI examinations and genetic factors with multivariate techniques from genetic analyses to investigate whether different anatomical subdivisions share common genetic factors. Based on the analysis of a large pediatric population, their work suggests that the great majority of variability in cerebrum, thalamus, cerebellum and basal ganglia is determined by a single genetic factor. Most of the variability in the corpus callosum was explained by additive genetic effects that were largely independent of other structures. Their work also observed small but significant environmental effects common to the thalamus, basal ganglia and lateral ventricles.

### Optimizing MRI brain imaging and analysis

MVA techniques can play a role in assessing imaging performance and optimizing image acquisition and its analysis. White et al. (2014) employed fuzzy set theory in a pediatric resting state fMRI study and determined that 5.5 min of resting state acquisition time was required to produce a stabilized set of brain network measurements. Zhou et al. (2011) demonstrated that deriving a Granger causality model from multivariate autoregressive models can yield greater accuracy in detecting network connectivity in fMRI examinations. Shehzad et al. (2014) developed a multivariate distance-based regression to assist in connectome-wide association studies and demonstrated it on healthy development data. Pontabry et al. (2013) developed a probabilistic approach to Q-ball imaging (an extension of DTI) tractography and demonstrated the technique on *in utero* fetal brain imaging examinations. They also demonstrated that their technique can outperform existing fiber tracking algorithms based on the Fiber Cup phantom challenge data (Pontabry et al., 2013). Goodlett et al. (2008) presented a framework for hypothesis testing of differences between DTI tractography pathways identified as bilateral cortico-spinal tracts and tracts running through the genu and splenium of the corpus callosum. Their approach incorporates PCA for signal smoothing. Multivariate discriminant analysis is performed on the PCA smoothed data and normalization is performed across a population with DTI atlas building procedures. Their methodology was evaluated on a pediatric study of 22 one-year-old and 30 two-year-old children.

### The effect of the senses on the brain

Sense organs (eyes, ears, etc.) contain concentrations of sensory neurons which can be stimulated by external physical effects (light, sound, etc.). These sensory neurons then stimulate additional neural activity in the brain. Considerable research has looked at the relationship between functional MR brain imaging and controlled sensory stimulation and a few of these studies have also incorporated MVA. Schopf et al. (2014) used fMRI technology to study fetal eye movements *in utero*. Their approach included the use of the random forest machine learning algorithm (Breiman, 2001) to classify whether individual pixels were part of the fetal eye. Their results indicate that the relationship between eye movement and vision develops before birth. Their work also incorporated ICA to identify networks showing the highest correlation with fetal eye movement. This included association related areas such as the angular gyrus, inferior parietal gyrus, the superior frontal gyrus and the medial occipital gyrus.

Jardri et al. (2008) investigated fetal cortical activation due to sound stimulus at 33 GW using fMRI examinations. Their approach included the use of ICA for processing fMRI data to assist in the identification of brain regions exhibiting similar activation patterns. This study involved a direct auditory stimulus applied to six pregnant women's abdomens. Standard univariate voxel-wise analysis demonstrated that two of the six subjects exhibited significant activation in the left temporal lobe. MVA using ICA demonstrated that three out of six subjects exhibited significant activation in the left temporal lobe, implying that MVA techniques have the potential to identify patterns of brain activation missed by traditional univariate analyses. In an additional study, Jardri et al. (2012) studied fetal response to auditory stimulation induced by maternal speech using fMRI and ICA. This analysis was performed *in utero* on subjects at 33 and 34 GW. Their results demonstrated left temporal lobe activation in each of three fetuses imaged without motion artifacts. The authors reported their results as representing the first *in vivo* evidence for the development of maternal voice recognition *in utero*.

## Discussion

The results of this systematic review demonstrate a wide variety of applications of multivariate analysis (MVA) techniques applied to brain MRI examinations of healthy pediatric, neonatal and fetal populations. Since the ideal combination of MVA technique and medical imaging derived clinical information is unknown *a priori*, an enormous amount of research is required to fully optimize MVA's potential in this domain. Recent years have exhibited ample growth in the application of MVA techniques with ongoing growth expected. However, substantial challenges will accompany future research in this domain.

There are substantial strengths and weaknesses of the wide variety of multivariate analysis techniques available. Feature reduction techniques (such as PCA and ICA) can be useful in finding latent factors that can represent underlying physiological conditions that can only be assessed by acquiring a multitude of measurements. These techniques have considerable overlap with the functionality of machine learning algorithms. In supervised learning, the support vector machine (SVM) has shown itself to be a high performing robust learning algorithm which is particularly resilient to situations where it is provided with small numbers of training samples. The related relevance vector machine has been shown to produce learning solutions that rely on fewer training samples than the SVM allowing prediction to be computed more efficiently. Linear discriminant analysis is a classical learning technique which can reliably perform pattern recognition but often underperforms techniques like the SVM. The artificial neural network's strength lies in modeling the learning abilities of the human brain which is unmatched by current machine learning technology. However, artificial neural networks typically attempt to model a learning problem with far fewer neurons than the task would likely need in order to be accomplished in the human brain. The random forest is a high performing algorithm that explores a wide variety of possible decisions that could lead to accurate predictions and is particularly capable of exploiting feature measurements with limited separation information embedded therein. Finally, it should be noted that machine learning technology is too often treated as a black box whose internal behavior is unknown to the researcher. The technologies available provide the ability to analyse their behavior, a task that all pattern recognition researchers should engage in, not only to understand the nature of the technology they've developed, but also to understand the physiological significance of the patterns that the technology exploits in order to make its predictions.

One major obstacle in this application domain is caused by patient motion which is particularly challenging in pediatrics because children tend to have a harder time remaining still during imaging. Children asked to remain still in the scanner may forget over the course of the examination. Image registration is a class of technology used to compensate for patient motion but this is a challenging problem for which there is no accepted gold standard solution. Registration to standard templates (or brain atlases) is typically based on adult brains (Talairach and Tournoux, 1988) and it has been shown that normalization procedures used can cause distortions in the brain examinations of children 6 years old and under (Muzik et al., 2000). Distortional effects may adversely affect MVA results and so care should be taken to avoid providing data exhibiting distortional artifacts to MVA technology.

While feature selection can be addressed as a class of technology independent of supervised learning, some supervised learning techniques incorporate feature selection while others do not. Feature selection is particularly important in the characterization of neurodevelopment in a fetal, neonatal and pediatric population because we acquire a multitude of measurements distributed across the brain and we don't know *a priori* all the brain regions that should be included in a given analysis. The same is also true for identifying brain regions whose structure or function at a particular time point is critical in a future phase of healthy brain development, thus feature selection technologies have considerable potential in improving our understanding of healthy brain development. Feature selection research could lead to new technologies to assist in the characterization, detection and diagnosis of a variety of medical conditions as aberrations from expected healthy growth patterns.

DTI and tractography are critical tools to assess maturation of brain development, especially emerging or regressing tissue coherency. However, limited MVA research has been conducted incorporating diffusion imaging examinations as compared with T1- or T2-weighted MRI. This may be because of a variety of factors including the complexity of interpretation and analysis of DTI data with multiple directions of tissue coherency, higher sensitivity to motion, higher dependencies on SNR, reliability of tractography reconstruction and DTI's much more limited routine clinical use. However, considerable MVA research incorporating DTI is under development and exciting advances in this field are expected in the next few years that combine advanced MVA technologies with the enormous amounts of data acquired in DTI examinations.

The scientific literature has seen enormous growth in studies focused on developmental imaging of pre-adult populations that make use of MVA technologies. However, much of this work has been focused in pediatric imaging with considerably less focus on neonatal and fetal populations. Neonatal imaging is more challenging as brain size is considerably smaller than in pediatrics and it is more challenging for imaging technicians to get a neonate to remain motionless during their imaging examination. Patient movement induces multiple types of imaging artifacts which can negatively affect MVA. Fetal imaging is the largest challenge of the three as the brain sizes are the smallest and movement remains a major issue. Furthermore, MRI technology is reliant on the spatial proximity of a coil/antenna to the tissue/organ being imaged. Normal brain imaging benefits from a specialized head coil that is mounted immediately adjacent to the subject's cranium, however, in fetal imaging this is not possible and so coils are located outside the mother's abdomen, inherently reducing image quality. Additional challenges exist in fetal imaging due to *in utero* variations in tissue contrast relative to that observed at later developmental stages. Regardless of the many challenges inherent in the use of MVA in the imaging of fetal, neonatal and pediatric populations, there is considerably large potential for ongoing growth in this research field.

## Conclusion

Multivariate analysis (MVA) technologies can play a useful role in helping to answer questions about structural and functional organization in the developing brain. Furthermore, MVA techniques have the potential to better characterize subject anatomy and physiology than a univariate technique could produce alone. MVA technologies have tremendous potential in the creation of the next generation of clinical diagnostic tests informed by the large amount of information acquired by MRI. MVA technologies have exhibited enormous growth in developmental brain MRI in pre-adult populations with an emphasis on pediatric imaging. The technologies are very flexible and a wide range of potential applications have already been investigated, however, so many variations on MVA technologies are available in the scientific literature that ample research will need to be performed in order to properly evaluate the trade-offs imposed by the selection of a given MVA technique for any particular analysis task. Future work will look at improving MVA techniques and adapting them to better characterize healthy neurological development in pre-adult populations.

### Conflict of interest statement

The authors declare that the research was conducted in the absence of any commercial or financial relationships that could be construed as a potential conflict of interest.
